# The interplay between hospital and surgeon factors and the use of sentinel lymph node biopsy for breast cancer

**DOI:** 10.1097/MD.0000000000004392

**Published:** 2016-08-07

**Authors:** Tina W.F. Yen, Jianing Li, Rodney A. Sparapani, Purushuttom W. Laud, Ann B. Nattinger

**Affiliations:** a Department of Surgery, Division of Surgical Oncology; b Division of Biostatistics; c Department of Medicine; d Center for Patient Care and Outcomes Research, Medical College of Wisconsin, Milwaukee, WI, USA.

**Keywords:** breast cancer, hospital characteristics, sentinel lymph node biopsy, surgeon characteristics

## Abstract

**Background:**

Several surgeon characteristics are associated with the use of sentinel lymph node biopsy (SLNB) for breast cancer. No studies have systematically examined the relative contribution of both surgeon and hospital factors on receipt of SLNB.

**Objective:**

To evaluate the relationship between surgeon and hospital characteristics, including a novel claims-based classification of hospital commitment to cancer care (HC), and receipt of SLNB for breast cancer, a marker of quality care.

**Data Sources/Study Design:**

Observational prospective survey study was performed in a population-based cohort of Medicare beneficiaries who underwent incident invasive breast cancer surgery, linked to Medicare claims, state tumor registries, American Hospital Association Annual Survey Database, and American Medical Association Physician Masterfile. Multiple logistic regression models determined surgeon and hospital characteristics that were predictors of SLNB.

**Results:**

Of the 1703 women treated at 471 different hospitals by 947 different surgeons, 65% underwent an initial SLNB. Eleven percent of hospitals were high-volume and 58% had a high commitment to cancer care. In separate adjusted models, both high HC (odds ratio [OR] 1.53, 95% confidence interval [CI] 1.12–2.10) and high hospital volume (HV, OR 1.90, 95% CI 1.28–2.79) were associated with SLNB. Adding surgeon factors to a model including both HV and HC minimally modified the effect of high HC (OR 1.34, 95% CI 0.95–1.88) but significantly weakened the effect of high HV (OR 1.25, 95% CI 0.82–1.90). Surgeon characteristics (higher volume and percentage of breast cancer cases) remained strong independent predictors of SLNB, even when controlling for various hospital characteristics.

**Conclusions:**

Hospital factors are associated with receipt of SLNB but surgeon factors have a stronger association. Since regionalization of breast cancer care in the U.S. is unlikely to occur, efforts to improve the surgical care and outcomes of breast cancer patients must focus on optimizing patient access to SLNB by ensuring hospitals have the necessary resources and training to perform SLNB, staffing hospitals with surgeons who specialize/focus in breast cancer and referring patients who do not have access to SLNB to an experienced center.

## 1 Introduction

Sentinel lymph node biopsy (SLNB) has become the standard of care for axillary staging in early stage, clinically node-negative breast cancer and is considered a quality measure.^[1–4]^ Compared with axillary lymph node dissection (ALND), which involves the removal of most lymph nodes, SLNB typically involves the removal of only a few lymph nodes and is associated with similar disease-free survival but a significantly reduced likelihood of developing lymphedema (arm swelling) and other arm/shoulder morbidity which are associated with decreased quality of life.^[5–9]^

Our group and others have demonstrated that women are more likely to undergo SLNB if they are treated by surgeons who specialize or focus in breast cancer, defined by volume or percentage of breast cancer cases and membership in breast and surgical oncology societies.^[10–12]^ However, the hospital where surgery is performed is an important potential facilitator of SLNB use, as hospital commitment is required to perform this complex process which requires an interdisciplinary collaboration among radiology, nuclear medicine, operating room staff, surgeons, and pathology.^[2]^

Prior studies have demonstrated that women who undergo treatment at hospitals affiliated with National Cancer Institute (NCI)-cooperative research networks or accredited American College of Surgeons (ACS) Commission on Cancer (CoC) programs are more likely to receive SLNB.^[13–16]^ Studies examining the relationship between hospital volume (HV) and teaching status and receipt of SLNB have yielded conflicting results.^[10,13,15,17]^ To our knowledge, no studies have examined the interplay between hospital and surgeon characteristics and the use of SLNB. Examining hospital factors, including a hospital's commitment to cancer care, is important, particularly when a hospital must commit significant capital investment and resources and ensure collaboration between disciplines to introduce a new cancer-specific technology or procedure.

In this study, we build upon our prior work and evaluate the relationship between hospital characteristics, including a hospital's focus/commitment to cancer care, and receipt of SLNB. We also explore the relative contributions of hospital and surgeon characteristics on the type of axillary surgery performed. We hypothesize that hospital factors contribute to receipt of SLNB but surgeon factors play a more important role.

## 2 Methods

### 2.1 Study cohort and data sources

This population-based cohort consists of participants in an NCI-sponsored survey study examining outcomes of breast cancer care. Elderly women from 4 geographically and racially diverse states (CA, FL, IL, and NY) were identified from Medicare claims as having undergone incident breast cancer surgery in 2003.^[18]^ Details regarding study recruitment, sample, and survey assessments have been previously described.^[12,19]^ The participation rate for the initial survey wave was 70%; nonparticipants were more likely to be older (75+ years) and reside in NY but participation did not differ based on socioeconomic status, race/ethnicity, comorbidity, or type of breast surgery.^[19]^ For this current study, the cohort consists of 1703 women with invasive breast cancer, validated by state tumor registry information,^[20]^ who underwent axillary surgery (SLNB or ALND), completed wave 2 of the survey and had hospital and surgeon information identifiable by Medicare claims.

Medicare claims information was collected from the denominator, inpatient, outpatient, and carrier files. The American Hospital Association Annual Survey Database contains over 800 fields of data on over 6500 United States (U.S.) hospitals.^[21]^ Surgeon characteristics were obtained from the American Medical Association Physician Masterfile,^[22]^ and membership in the Society of Surgical Oncology (SSO)^[23]^ and American Society of Breast Surgeons (ASBrS)^[24]^ was determined from online directories, as previously described.^[12]^

### 2.2 Variable definitions

Table 1 summarizes the hospital and surgeon characteristics examined and the source of information. Patient characteristics (age, race, state of residence, body mass index [BMI] at time of surgery, and comorbidity) and receipt of adjuvant treatments (radiation, chemotherapy, and hormonal therapy) were derived from Medicare claims or survey response (race, BMI, and adjuvant treatments).^[12]^ Comorbidity was determined from inpatient, outpatient, and carrier Medicare claims for the year preceding the incident breast cancer diagnosis based on the NCI Combined Comorbidity Index algorithm by Klabunde et al,^[25]^ which is specific for patients with breast cancer. Type of axillary surgery performed was determined from Medicare claims, as previously described.^[12,26–28]^ Patients were classified into 1 of 2 groups: initial SLNB, which included patients who underwent only SLNB and those who went on to completion ALND; and initial ALND.

**Table 1:**
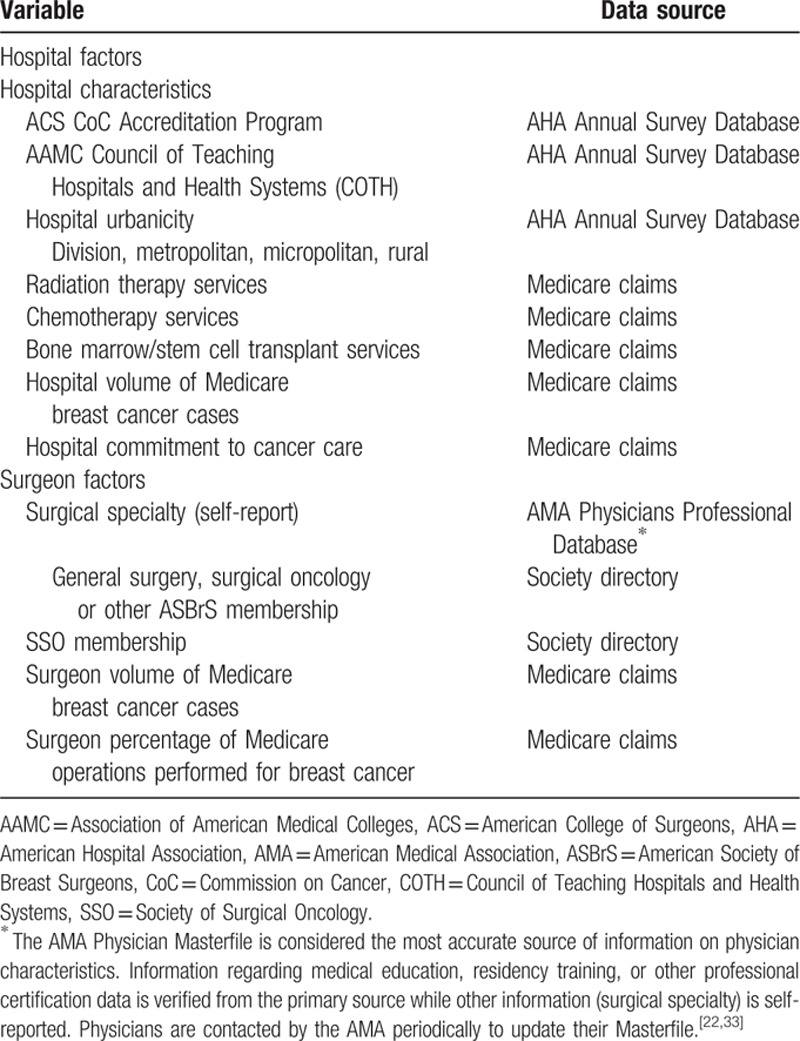
Hospital and surgeon variable definitions and data source.

### 2.3 Hospital characteristics

Hospitals evaluated in this study included only hospitals where the women in this cohort received breast cancer surgery. Provider hospital codes from Medicare claims were linked to the 2004 American Hospital Association Annual Survey Database to determine ACS CoC accreditation and Association of American Medical Colleges Council of Teaching Hospitals and Health Systems (COTH) status in 2003. Accreditation in the ACS CoC Program is voluntary and requires key elements related to clinical care, quality improvement, and maintenance of a cancer registry database.^[29]^ More than 70% of all newly diagnosed cancer patients in the U.S. are treated in approximately 1500 ACS CoC-accredited facilities. COTH membership includes approximately 400 of the nation's leading teaching hospitals and health systems and is recognized as a benchmark for excellence in patient care, research, and education.^[30]^ Hospital urbanicity (division, metropolitan, micropolitan, and rural) was determined by core-based statistical area, an established U.S. Census Bureau standard.^[31]^ Hospitals in an area of fewer than 10,000 were deemed “rural.” A “micropolitan” area has at least 1 urban cluster of 10,000 to 50,000 population. A “metropolitan” area has at least 1 core urban area of 50,000 or more inhabitants. If a metropolitan area contains a single core with a population of at least 2.5 million, the metropolitan area is subdivided into smaller “metropolitan divisions,” representing the most urban areas.

### 2.4 Determination of hospital case volume and hospital commitment to cancer care

Medicare claims were used to determine annual Medicare hospital case volume of breast cancer surgery cases, as previously described.^[32]^ Volume for each hospital was based on Medicare claims for all breast cancer cases treated in each state, not solely for cohort subjects. HV was categorized into 3 groups (low: 0–<20 cases per year; medium: 20–<40 cases per year; and high: 40 or more cases per year) with approximately equivalent number of patients in each group.

Medicare claims were used to determine if a hospital provided services for 4 high technology, cancer specific services: radiation therapy, including intensity-modulated radiation therapy (IMRT; a more specialized, targeted form of radiation therapy), chemotherapy, and bone marrow/stem cell transplantation.^[26–28]^ Radiation therapy services were defined by Current Procedural Terminology codes for a physician or outpatient claim for radiation treatment delivery, neutron beam treatment delivery, proton beam treatment delivery, or radiotherapy treatment management.^[27]^ Chemotherapy services were defined by Healthcare Common Procedure Coding System J codes for chemotherapeutic agents.^[28]^ Bone marrow/stem cell transplant services were defined by providing bone marrow or stem cell services/procedures, autologous bone marrow/stem cell transplantation, and allogeneic bone marrow/stem cell transplantation.^[26,27]^

A hospital's focus/commitment to cancer care was classified as low or high depending on ACS CoC accreditation status and whether the hospital billed for these 4 cancer specific services: bone marrow/stem cell transplantation, chemotherapy, radiation therapy, and IMRT. Hospitals meeting 2 or more of these 5 criteria were classified as having a high commitment to cancer care; the remaining hospitals were classified as having a low commitment to cancer care.

### 2.5 Surgeon characteristics

Provider codes from Medicare claims of the surgeons operating on cohort patients were linked to the 2004 American Medical Association Physicians Professional Database, which is considered the most accurate source of information on physician characteristics.^[22,33]^ All surgeon characteristics listed in Table 1 have been previously described.^[12]^ In brief, for each surgeon, Medicare claims were used to determine annual Medicare surgeon volume of breast cancer cases and the percentage of Medicare operations devoted to breast cancer cases.^[12,34]^ Surgeon percentage of practice devoted to breast cancer cases was determined by dividing the annual number of patients who underwent an initial breast cancer surgery by the number of patients who underwent a general surgery operation performed that same year. Both surgeon volume and the specialization measure were based on Medicare claims for all surgeries performed in each state, not solely for cohort subjects.

### 2.6 Statistical analysis

Multiple logistic regression models were developed to determine surgeon and hospital characteristics that were independently associated with the outcome, type of initial axillary surgery performed (SLNB or ALND). To account for clustering by surgeons and hospitals, random effects at both levels were included.^[35]^ Covariates controlled for in all models were determined a priori and included 4 patient characteristics (age, race, BMI at time of surgery, and comorbidity) as well as geographic location (state). Final pathologic tumor stage was not included as this information is not available until after surgery has been performed and therefore does not influence the type of axillary surgery performed.

To assess the independent effect of hospital characteristics on initial axillary surgery (Table 4), the 1st model included 4 hospital characteristics (HV of breast cancer cases, hospital commitment to cancer care [HC], COTH status, and urbanicity). Two additional hospital only models were run: one with HC, COTH status, and urbanicity; the other one with HV, COTH status, and urbanicity. To assess the independent effect of surgeon characteristics on initial axillary surgery, the next model included the 4 surgeon characteristics previously shown to be predictors of SLNB ^[12]^: surgeon percentage, surgeon volume, ASBrS and SSO membership, and the interaction term between surgeon percentage and surgeon volume (Table 5). To examine the relationship between hospital and surgeon characteristics, the final model included the 4 surgeon characteristics and 4 hospital characteristics. Estimates were calculated using the GLIMMIX procedure in SAS statistical software (Version 9.3, SAS Institute; Cary, NC). For statistical summaries, the odds ratios (ORs) of the fixed effects from these mixed effects logistic regression models are reported.^[35–37]^

This study was approved by the Centers for Medicare and Medicaid Services, each state Institutional Review Board, and our institution's Institutional Review Board.

## 3 Results

The characteristics of the study cohort are summarized in Table 2. The mean age of the 1703 women at the time of surgery was 72.7 years (SD 5.3), 92% were white. At the time of surgery, the majority (58%) was overweight or obese and 62% were healthy with an NCI combined comorbidity score of 0. On final pathology, 78% had invasive disease confined to the breast (Surveillance, Epidemiology, and End Results [SEER] summary stages 1–2), 22% were node-positive (SEER summary stages 3–5). Surgically, 63% underwent breast-conserving surgery, 65% underwent initial SLNB, and 35% had initial ALND. About two-thirds (68%) received radiation therapy, 25% chemotherapy, and 70% hormonal therapy.

**Table 2:**
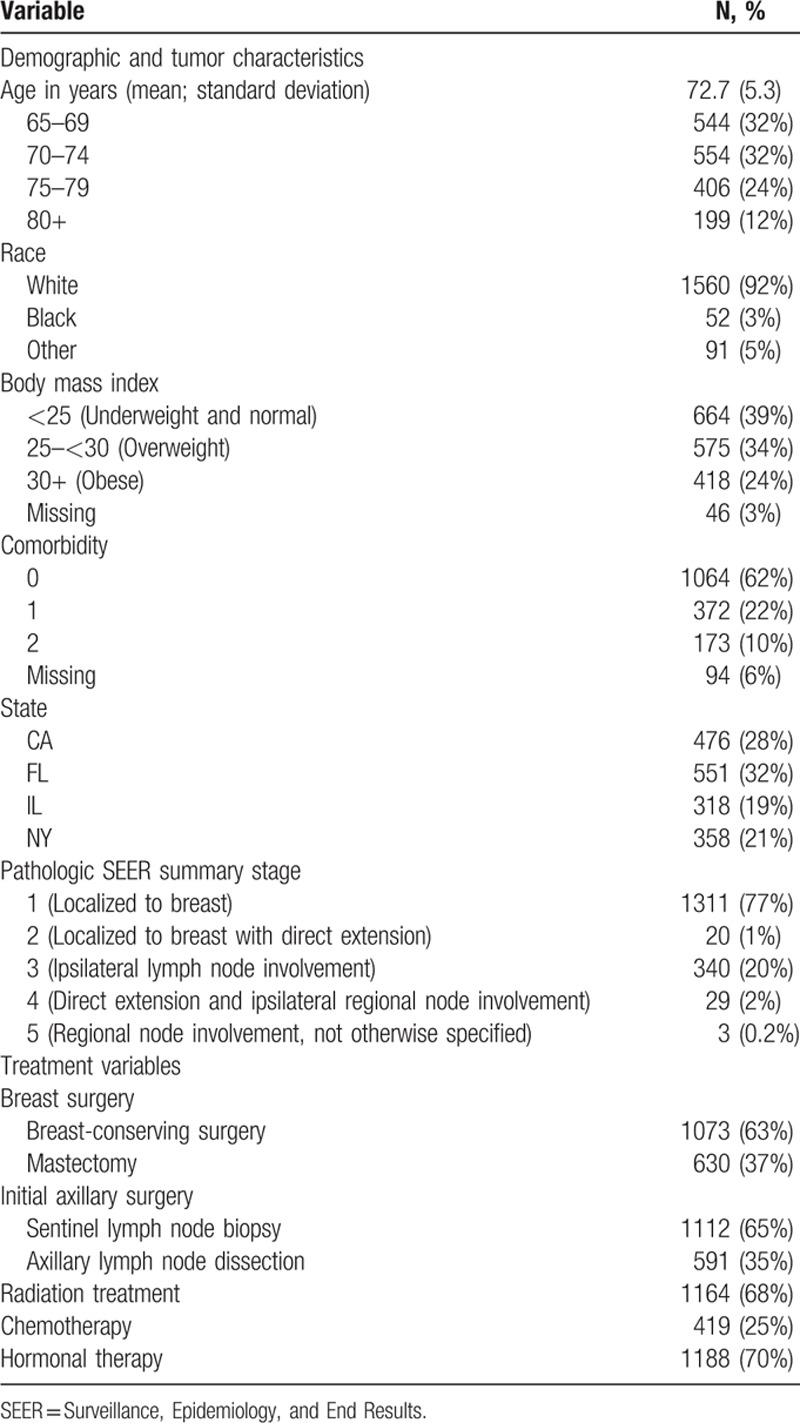
Characteristics of study cohort.

### 3.1 Hospital characteristics/measures

This cohort underwent surgery at 471 different hospitals (Table 3). More than half (53%) of hospitals were ACS CoC-accredited cancer programs; 73% provided chemotherapy services, 45% radiation services, and 27% IMRT. Only 6% provided bone marrow/stem cell transplantation services. HC was low for 41% and high for 59% of hospitals. Almost two-thirds (63%) were low-volume hospitals while 11% were high-volume hospitals; 12% were COTH teaching hospitals. Most hospitals (87%) were located in urban (division or metropolitan) areas.

**Table 3:**
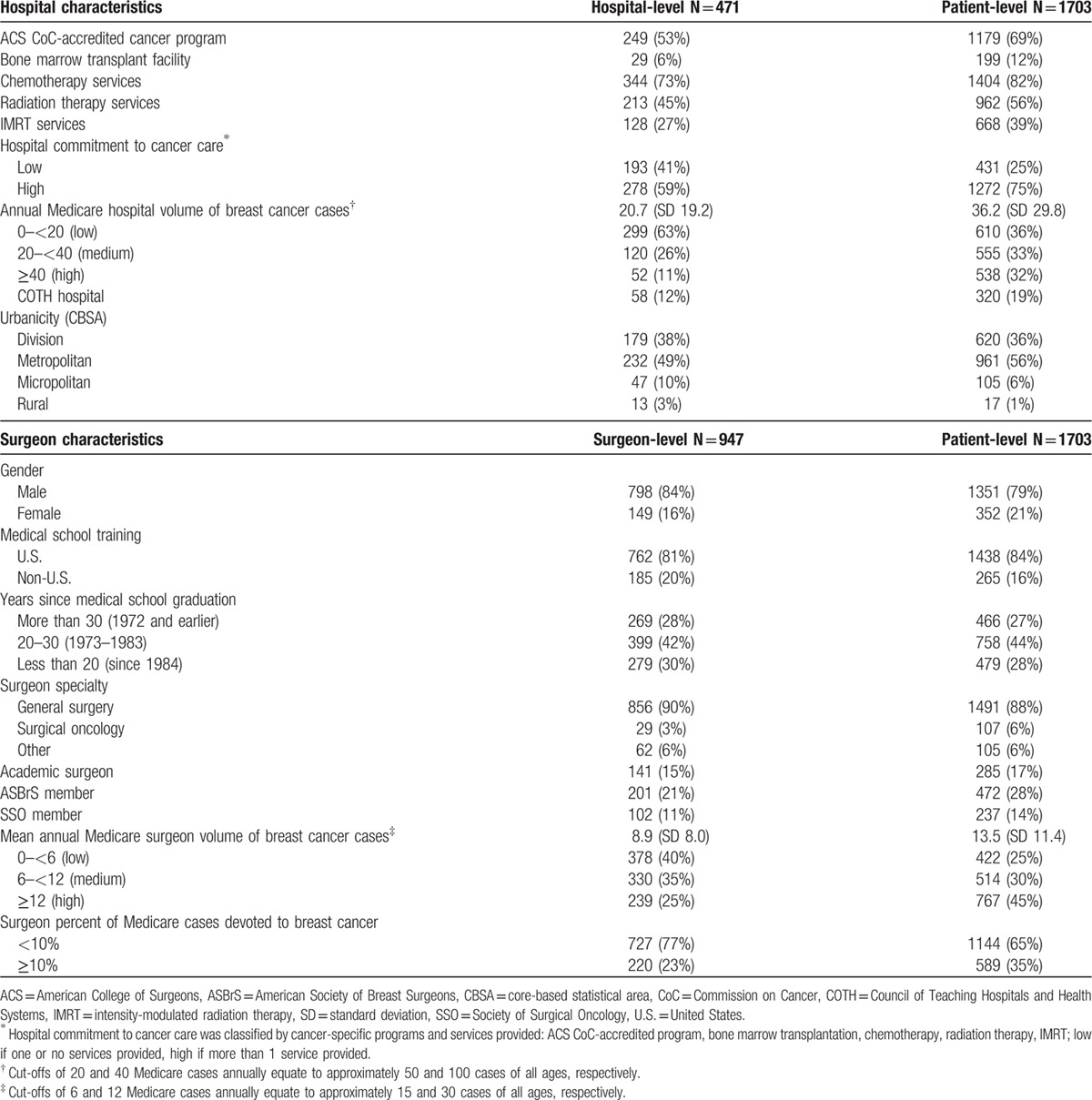
Distribution of hospital and surgeon characteristics.

At the patient level, 69% were treated at an ACS CoC-accredited hospital, 75% at a hospital with a high commitment to cancer care, 19% at COTH teaching hospitals, and 92% in hospitals in urban settings.

### 3.2 Surgeon characteristics

Overall, 947 different surgeons operated on these 1703 women (Table 3). The majority (90%) were general surgeons, only 3% were surgical oncologists. Less than a quarter (21%) were ASBrS members and 11% were SSO members. Forty percent were low-volume surgeons, performing fewer than 6 Medicare cases annually, which equates to approximately 15 cases in women of all ages; 25% were high-volume surgeons, performing 12 or more Medicare cases annually, which equates to 30 or more cases in women of all ages. Less than a quarter (23%) devoted at least 10% of their cases to breast cancer.

At the patient level, 25% underwent an operation by a low-volume surgeon while 45% were treated by a high-volume surgeon. One-third (35%) were operated on by surgeons who devoted at least 10% of their operations to breast cancer. Overall, 28% and 14% were operated on by surgeons who were ASBrS or SSO members, respectively.

### 3.3 Effect of hospital characteristics on type of axillary surgery

Table 4 summarizes the results of 3 models that examine the relationship of hospital characteristics and type of axillary surgery performed. The model on the left includes both HC and HV. Compared to women treated at low-volume hospitals, women treated at high-volume hospitals had a higher likelihood of receiving SLNB (OR 1.66; 95% confidence interval [CI] 1.10–2.52). There was a trend toward women treated at hospitals with a high commitment to cancer care being more likely to receive SLNB (OR 1.36, 95% CI 0.97–1.92). Women residing in nonurban areas were significantly less likely to receive SLNB (OR 0.43, 95% CI 0.25–0.74). Older age, presence of comorbidities, and black race were associated with a lower likelihood of receiving SLNB. There was no interaction between HV and HC (*P* = 0.91). Overall, the model's predictive concordance was reflected in the c-statistic of 0.67.

**Table 4:**
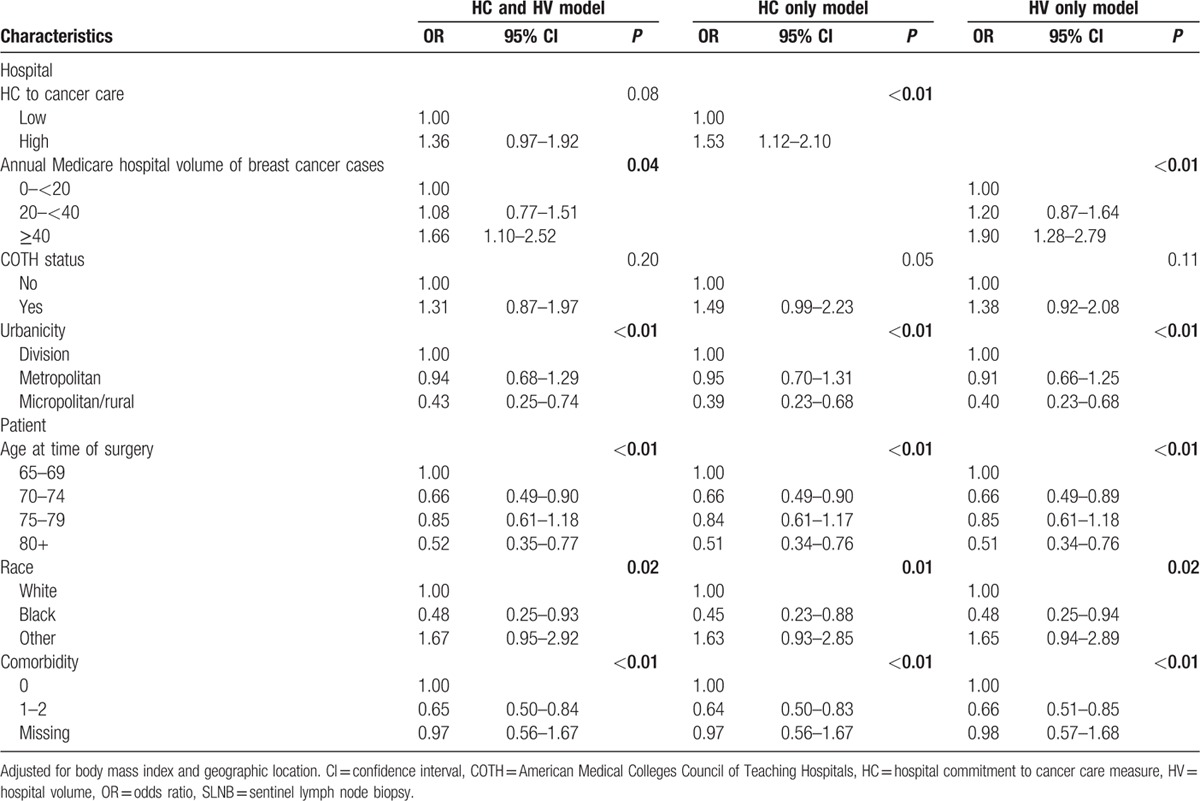
Mixed-effects models associating hospital characteristics with receipt of initial SLNB.

If the model is run with HC but not HV (middle column of Table 4; c-statistic 0.66), being treated at a hospital with a high commitment to cancer care is significantly associated with receiving SLNB (OR 1.53; 95% CI 1.12–2.10). If the model is run with HV but not HC (right column; c-statistic 0.66), being treated at a high volume hospital is associated with receipt of SLNB (OR 1.90; 95% CI 1.28–2.79). Since the c-statistics are similar for the last 2 models, individually, HC and HV have similar abilities to predict the likelihood of receiving SLNB.

### 3.4 Combined effect of surgeon and hospital characteristics on type of axillary surgery

The left side of Table 5 displays the hospital model results shown in Table 4 that includes both HC and HV. The middle column of Table 5 displays the surgeon characteristics model. Consistent with our prior work in a similar cohort from this survey study,^[12]^ women operated on by surgeons who had a higher volume and/or percentage of breast cancer cases were more likely to undergo SLNB; an interaction exists between these 2 variables (*P* = 0.03). Women operated on by surgeons who were ASBrS or SSO members were also more likely to undergo SLNB.

**Table 5:**
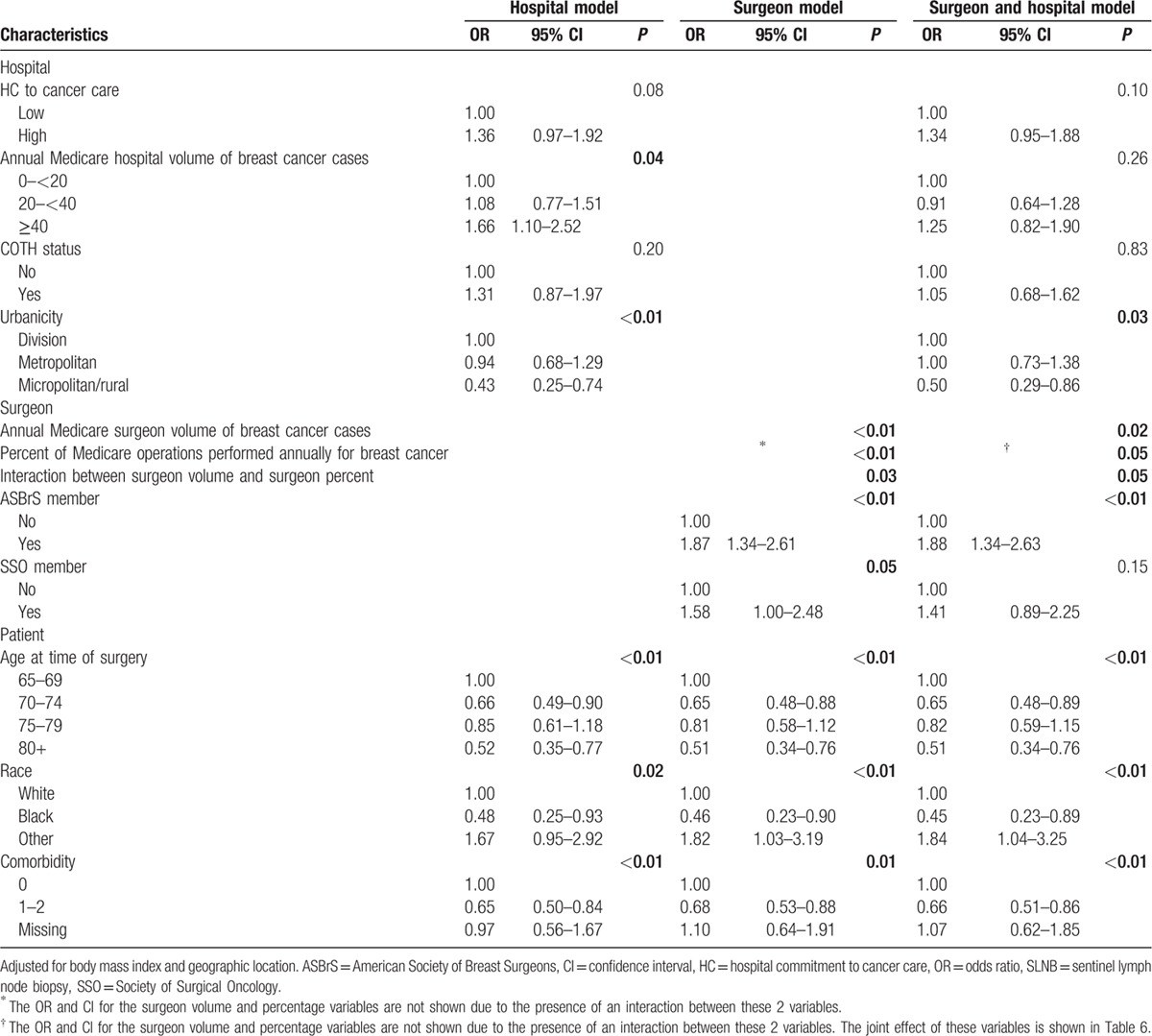
Mixed effects models for receipt of initial SLNB.

The right side of Table 5 summarizes the combined effect of both surgeon and hospital characteristics on the type of axillary surgery performed. Adding the surgeon characteristics to the hospital model minimally modified the effect of high HC (OR 1.34, 95% CI 0.95–1.88) but significantly weakened the effect of HV (*P* = 0.26), particularly the effect of high HV. In the combined model, the strength of the association between 3 surgeon characteristics (higher volume and higher percentage of breast cancer cases and ASBrS membership) and the likelihood of receiving SLNB persisted despite the addition of hospital characteristics. There was no evidence of collinearity, as judged by variance inflation factor and stability of standard errors (data not shown for brevity), or an interaction between HV and HC (*P* = 0.81) or between surgeon volume and HV (*P* = 0.46).

In the combined surgeon and hospital characteristics model, the interaction between surgeon volume and percentage of breast cancer cases persisted (*P* = 0.05), and the interaction effect (Table 6) is consistent with our prior work.^[12]^ The surgeon volume effect was highest among surgeons with lower percentages of breast cancer cases. Among women operated on by surgeons who devoted less than 10% of their operations to breast cancer cases, if their surgeon had a higher volume of cases, they were more likely to receive SLNB (OR 1.67 for high volume; OR 1.43 for middle volume), compared to a low-volume and low-percentage surgeon. Among women operated on by surgeons who devote at least 10% of their operations to breast cancer, there was no effect of surgeon volume on the likelihood of receiving SLNB.

**Table 6:**
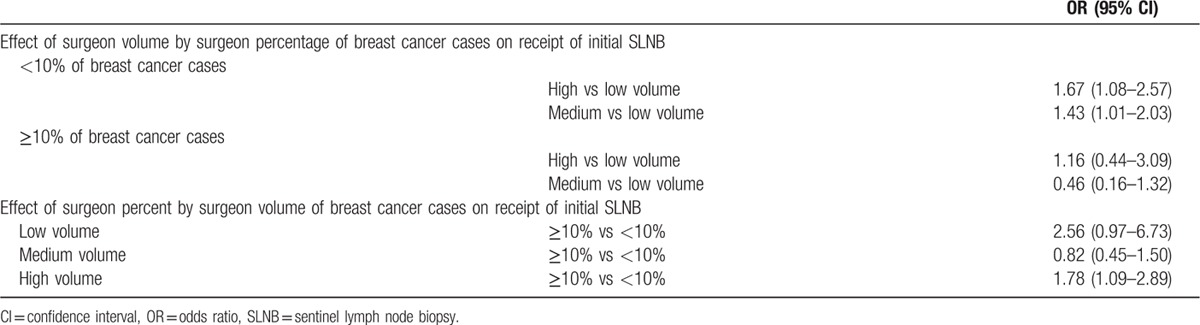
Interaction effect between surgeon volume and surgeon percent of breast cancer cases and receipt of initial SLNB.

Even more pronounced was the effect of surgeon percentage of breast cancer cases, particularly among surgeons with lower volumes of breast cancer cases. Among women operated on by low-volume surgeons, if their surgeon devoted at least 10% of their operations to breast cancer, they had 2.56-fold odds of receiving SLNB, compared to a low-volume and low-percentage surgeon. Among women operated on by high-volume surgeons, if their surgeon devoted at least 10% of their operations to breast cancer, they had 1.78-folds odds of receiving SLNB, compared to a high-volume but low-percentage surgeon.

## 4 Discussion

In this population-based cohort of over 1700 older breast cancer survivors who all underwent axillary surgery, 65% underwent an initial SLNB. The majority (59%) of hospitals were classified as having a high commitment to cancer care and treated 75% of the patients. Only 11% were high-volume hospitals and 87% were located in urban settings. In the model examining the effects of hospital characteristics, patients treated at high-volume hospitals were more likely to receive SLNB and there was a trend toward women treated at hospitals with a high commitment to cancer care being more likely to receive SLNB. Consistent with prior studies, women were less likely to receive SLNB if they were older, black, had comorbidities, or lived in rural settings.^[13–17,38,39]^ Of note, despite the limited sample size of black women (n = 52) in this cohort, the negative effect of black race on receipt of SLNB was substantial.

When surgeon characteristics were added to this model, the magnitude of the HV effect was less and no longer significant. This finding is likely explained by the high HV effect seen in the hospital characteristics only model being mediated by high-volume and/or high-percentage surgeons working at high-volume hospitals. Interestingly, the HC effect remained relatively stable and borderline. The large negative effect of nonurban hospital location persisted, likely explained by the lack of resources or ability to perform SLNB in these settings, as this procedure requires a hospital's commitment of numerous disciplines and resources, including a nuclear medicine facility and the logistics and strict regulatory requirements of radioisotope use, to be successful. Several surgeon characteristics (higher volume and higher percentage of breast cancer cases, membership in ASBrS) remained significant strong predictors of receiving SLNB, even when controlling for various hospital characteristics. Therefore, even though the resources to perform SLNB are determined at the hospital level, the most important predictors of whether a patient receives SLNB are related to the treating surgeon's expertise/experience in breast cancer care. These findings support the conclusions of a recent systematic review that higher surgeon volume and specialization for a variety of different surgeries are associated with improved patient outcomes, while higher HV is of limited benefit.^[40]^

To our knowledge, this is the first study to assess a hospital's commitment or focus in cancer care by using a novel, claims-based assessment of cancer-specific (but not cancer disease-specific) services that are provided by a hospital: bone marrow/stem cell transplantation, chemotherapy, radiation therapy, and IMRT. A hospital's focus/commitment to cancer care was classified as low or high depending on ACS CoC accreditation status and whether the hospital billed for any of the 4 cancer specific services. Given the possibility that self-reported survey information about hospital services actually performed may not be completely accurate, we used billing claims data to objectively determine whether these services were actually provided by each hospital. We elected to include only high technology, cancer specific treatment services that require significant hospital capital investment, infrastructure, and resources. We demonstrate that this relatively simple and objective assessment of a hospital's commitment to cancer care performed similarly to HV of breast cancer cases in predicting the likelihood of receiving SLNB.

Our study has several limitations inherent to observational research. First, it is possible that misclassification of the outcome (SLNB or ALND) occurred as this axillary surgery variable was based on claims data.^[12]^ However, Medicare billing claims are generally accurate for breast cancer surgical billing codes.^[41,42]^ Second, since information on clinical tumor stage determined before surgery was not consistently recorded in the state tumor registries, we could not conclusively determine which patients were appropriate candidates for SLNB. However, clinical stage of disease, especially early-stage disease, is not likely to vary systematically by surgeon or hospital characteristics. Third, because this cohort was confined to Medicare patients treated during a period when SLNB was considered an option to ALND for women with early-stage breast cancer, our findings may not be generalizable to younger or more contemporary populations.^[17,39,43–45]^

The fact that all patients were Medicare beneficiaries is a strength of the study as we could identify both hospitals and surgeons based on claims information, calculate HV, surgeon volume, and percentages of breast cancer cases, and perform our analyses of various hospital and surgeon characteristics by linkage with multiple other sources. This population-based cohort provided us with a unique opportunity to examine the relative contribution of both hospital and surgeon factors on the type of axillary surgery performed in real world practice. In addition, performing this study during the period of SLNB adoption allowed us to have a relatively heterogeneous cohort to examine the outcome of interest (65% SLNB vs 35% ALND). Although SLNB has become standard of care for axillary staging in early stage, clinically node-negative breast cancer, there are still about a quarter of women in more contemporary, population-based cohorts who are not appropriately receiving SLNB.^[38,46]^ In one SEER study examining patients with invasive breast cancer who underwent axillary surgery and were confirmed to be pathologically node-negative, only 73% received SLNB in 2008.^[38]^ Thus, not all women who are candidates for SLNB are receiving it, likely partially due to incomplete uptake by hospitals because of the logistical and legislative issues and expenses associated with radioisotope usage and maintenance of a nuclear medicine facility. Given the clear benefits of SLNB, particularly a reduced likelihood of developing lymphedema and other arm/shoulder morbidity that result in decreased quality of life and higher medical costs,^[5–9,47]^ it is imperative that access to SLNB continue to be improved. The surgeon and hospital factors that we identified as associated with receipt of SLNB in our study are modifiable factors that remain relevant today and could be used to further optimize access to SLNB.

In summary, in this geographically diverse population-based study of breast cancer survivors operated on predominately in the community setting, about two-thirds underwent an initial SLNB. Hospital factors were associated with SLNB receipt but surgeon factors (volume and specialization/focus in breast cancer) were more strongly associated. Since regionalization of breast cancer care in the U.S. is unlikely to occur due to logistical, geographic, financial, and patient barriers, efforts to improve the surgical care and outcomes of breast cancer patients must focus on optimizing patient access to SLNB by ensuring hospitals have the necessary resources and training to perform SLNB, staffing hospitals with surgeons who specialize/focus in breast cancer and referring patients who do not have access to SLNB to an experienced center.
