# The interplay between hospital and surgeon factors and the use of sentinel lymph node biopsy for breast cancer: Erratum

**DOI:** 10.1097/01.md.0000505544.39976.a7

**Published:** 2016-10-21

In the article “The interplay between hospital and surgeon factors and the use of sentinel lymph node biopsy for breast cancer”,^[1]^ which appeared in Volume 95, Issue 31 of *Medicine*, Table 1 was originally formatted incorrectly. The article has since been corrected online.

## References

[1] Yen TW Li J Sparapani RA The interplay between hospital and surgeon factors and the use of sentinel lymph node biopsy for breast cancer. *Medicine*. 2016 95:e4392. 27495053 10.1097/MD.0000000000004392 PMC4979807

